# Editorial: Microbe assisted plant resistance to abiotic stresses

**DOI:** 10.3389/fpls.2023.1277682

**Published:** 2023-09-01

**Authors:** Delong Meng, Mengting Maggie Yuan, Juan Li

**Affiliations:** ^1^ School of Minerals Processing and Bioengineering, Central South University, Changsha, China; ^2^ Key Laboratory of Biometallurgy, Ministry of Education, Changsha, China; ^3^ Department of Environmental Science, Policy, and Management, University of California, Berkeley, Berkeley, CA, United States; ^4^ College of Agronomy, Hunan Agricultural University, Changsha, China

**Keywords:** abiotic stress, plant resistance, plant microbiome, plant-microbe interaction, bioinformatics

The interaction between microorganisms and plants has emerged as a prominent research area in both microbiology and plant biology. Abiotic stresses, including drought, salinity, and heavy metal, exert substantial impacts on plant growth across the globe. These stressors, whether occurring individually or in combination, can disrupt nutrient uptake and hinder overall plant development (Mushtaq et al., 2023). However, beneficial microbes has shown potential in enhancing plant resilience to such abiotic challenges (Cardarelli et al., 2022; El-Shamy et al., 2022). Certain microorganisms inhabiting the rhizosphere and phyllosphere can facilitate plant water and nutrient acquisition while providing protection against harmful environmental toxins (Degani, 2021; Redondo et al., 2022).

The past decade has witnessed remarkable strides driven by advancements in sequencing and omic technologies, unraveling the intricate mechanisms governing plant-microbe interactions amidst abiotic stresses. These nuanced relationships are progressively being deciphered, providing insights that pave the way for predictive and modulatory strategies. Leveraging plant-microbe interactions to bolster plant adaptation to abiotic stresses holds transformative potential across agricultural productivity, bioremediation strategies, and ecological sustainability.

This research endeavor is aimed at spotlighting the essential role of microorganisms in bolstering plant resistance against abiotic stresses. Within this Research Topic, ten scholarly contributions delve into a variety of mechanisms by which microorganisms assist plants in adapting to environmental fluctuations, safeguarding their growth and survival. The inquiry also delves into the intricate impact of inter-root microbial communities on the broader health of plants. Collectively, these articles provide a comprehensive perspective on how microorganisms contribute to ecosystem functions and the well-being of plants.

Enhancing plant production and survival in response to urgent market demands and severe abiotic stresses has become a central focus of research. Qi et al. utilized RNA interference (RNAi) technology to construct an ihpRNA plant expression vector of the oleic acid desaturase (FAD2) gene, resulting in an elevated oleic acid content and a reduction in levels of linoleic acid and linolenic acid in rapeseed. Notably, the rhizosphere microbial community, employed as an indicator for the safety assessment of genetically modified plants, remained largely unaffected. This study presents a secure and effective method for enhancing plant production. Fu et al. focused on the impact of high-altitude environments on the endophytic microbial community of Ginkgo biloba. They revealed that altitude could regulate the microbial community structure by modulating Ginkgo biloba metabolites (flavonoids). This research unveiled the intricate interaction between microorganisms and plants, enhancing plant environmental adaptability. Li et al. investigated horizontal gene transfer (HGT) in plant genomes, identifying 235 genes from microbes that conferred abiotic stress resistance. These genes provided plants with defenses against various stressors such as toxic metals, drought, heat, and pollutants. Phylogenetic analysis supported the microbial origin of these genes. The research highlighted the crucial contribution of HGT to enhancing plants’ ability to cope with environmental challenges. Moreover, Wang et al. conducted an analysis of the composition and assembly of the microbial community in tobacco leaves. This study aimed to investigate the effects of summer climate and wildfire disease stresses on microorganisms. Their findings shed light on the significant role played by microorganisms in shaping the spatial and temporal patterns, processes, and response mechanisms of plants in their adaptation to environmental stresses. Collectively, these studies promise to not only deepen our comprehension of the contribution of microorganisms to plant adaptation under abiotic stresses, but also to unravel the intricate complexity of the symbiotic relationship between microorganisms and plants.

Plant rhizosphere microorganisms, as essential components of the plant holobiont, play a pivotal role in maintaining plant health and adaptability. This Research Topic illuminated the complex interaction between rhizosphere microbes and the overall health of plants. In the context of collaborative responses to plant diseases, Tang et al. investigated how tobacco bacterial wilt and black shank pathogens modified interactions within the rhizosphere microbial community. At the metabolic level, Actinobacteria strains that secreted antibiotics demonstrated effective pathogen inhibition, underscoring the significance of rhizosphere microbes in sustaining plant health. Similarly, Tao et al. explored how bacterial wilt disease impacts the root-associated microbiomes of tobacco plants. They found that the disease led to distinct changes in the microbiomes of different root compartments. The study highlighted the recruitment of beneficial bacteria and varying sensitivities among root areas. The research provides valuable insights into the responses of root-associated microbiomes to plant diseases. Poria et al. proposed a sustainable solution for remediating marginal land using Plant Growth-Promoting Bacteria (PGPB). They highlighted the role of plants and microorganisms in restoring soil quality in challenging environments. The review covered different phytoremediation methods, focusing on economically important plants and the benefits of PGPB in enhancing plant growth and bioremediation. The authors suggested using PGPB with dual precession technology to improve soil fertility and restore agriculture and native vegetation on marginal lands. In the realm of heavy metal remediation, Xiao et al. investigated how rhizosphere bacteria assisted in phytoremediation within a lead-zinc mining area. They compared the metal accumulation abilities of various plants and identified specific bacteria that influenced metal uptake and soil parameters. Their findings revealed a strong correlation between the composition of plant rhizosphere microorganisms and the plants’ capability to absorb heavy metals. The research provided insights into selecting plants for diverse metal remediation scenarios and emphasized the potential of rhizosphere bacteria to enhance multi-metal phytoremediation. Zakrzewska et al. used *Ochrobactrum* sp. POC9 metabolites to counter cadmium toxicity in soil. They investigated the impact of supplementing soil with carbonates-containing metabolites (MCC) on Cd mobility, plant growth, and uptake using *Petroselinum crispum*. MCC acted as a stabilizer for Cd and a stimulant for soil and plants. Li et al. investigated the interplay between rice plant rhizosphere microorganisms and cadmium uptake. The study unveiled significant impacts of specific bacterial strains’ inoculation on rice plant cadmium uptake and root endophytic bacterial communities. Furthermore, correlations were identified between the relative abundance of specific bacteria, cadmium content in rice plants, and cadmium enrichment coefficients in roots. These findings underscored the considerable potential of microorganisms in enhancing plant health and promoting sustainable agriculture. In sum, these studies not only deepen our comprehension of the profound connection between rhizosphere microbes and plant health but also unveil the diverse roles of microbes in plant adaptation and restoration capability.

Articles in this Research Topic not only uncovered the significance of microorganisms in aiding plants’ adaptation to abiotic stressors and the essential role of rhizosphere microorganisms in plant well-being, but also provide valuable guidance for achieving sustainable agricultural development and ecological preservation. By intensifying in-depth research into the interplay between microorganisms and plants, we aspire to better utilize these interactive relationships in the future, contributing to the sustainable development.

## Author contributions

DM: Writing – original draft, Writing – review & editing. MY: Writing – review & editing. JL: Writing – review & editing.
